# Self-Identity Development among Indigenous Adolescents from the Far North of Russia

**DOI:** 10.3390/bs9100106

**Published:** 2019-10-02

**Authors:** Natalia Flotskaya, Svetlana Bulanova, Maria Ponomareva, Nikolay Flotskiy, Tatiana Konopleva

**Affiliations:** Higher School of Psychology, Pedagogy and Physical Education Northern (Arctic) Federal University Named after M.V. Lomonosov, Arkhangelsk 163002, Russia

**Keywords:** self-identity, self-identity components, adolescents, Nenets

## Abstract

Self-identity is one of the fundamental needs of an individual. The most significant period of a person’s self-identity development is adolescence. The aim of this empirical study was to examine self-identity development among indigenous adolescents from the subarctic region of Russia. We identified specific features of personal identity among Nenets adolescents as compared to Russian adolescents. We also identified the dynamics of self-identity components among Nenets girls and boys during the transition period from the age of 12–13 to 14–15 years, and discovered characteristic features of self-identity among Nenets girls and boys.

## 1. Introduction

Modern socio-economic, cultural, and socio-educational environments do not always provide conditions for the development of a person’s self-identity; however, self-identity is a fundamental need of individuals, and it is necessary for their mental health, psychological integrity, and stability.

It is typical for modern psychology to interpret individual’s self-identity as a set of characteristics that distinguish one person from another and to define self-identity itself as a current state, namely the current perception of oneself at a certain stage in life. Self-identity develops over a lifetime and is accompanied by crises, that is, conflicts between the established identity and the current social situation [1,2]. Self-identity is a combination of such characteristics of a person as identity, integrity, and a unique structure that develops and evolves as a result of adaptation and reorientation in the constantly changing and transforming environment. Furthermore, identity is an expression of internal processes. It is a complex psychological phenomenon that integrates a set of ideas about the self, value orientations, and motivational components, and confirms an individual’s personality as a whole [3,4,5,6,7]. Researchers point out the dynamic nature and continuity of the identity development process, with adolescence being the point where a certain integrity of the identity development is reached ([8,9,10,11,12,13,14] and others).

Currently, psychologists around the world as well as in Russia are starting to study the characteristic features of self-identity development at different life stages. Adolescence is important for the psychological analysis of self-identity, since this period is characterized by a high intensity of the socialization and individualization processes [15]. Uncertainty brings many changes to the identity of modern adolescents, who are especially sensitive to social and value transformations, and it has a serious impact on their adaptive capacity, socialization, and future life prospects. Contemporary studies present various aspects of the psychological analysis of self-identity development in adolescence ([16,17,18,19] and others).

In our opinion, the study of self-identity among indigenous adolescents from the North is of particular interest, since their mentality is characterized by a peculiar duality in their view of life: the mythological consciousness and uniqueness of the traditional way of life on the one hand, and a special position in the system of relations with the dominant majority of the population on the other hand [20]. There is a number of studies exploring certain aspects of self-identity among adolescents from different ethnic groups living in Russia. Mironov, for instance, analyzed distinctive aspects of the attitude to the self among Ob-Ugric adolescents [21]. Nafanailova described self-identity among Sakha representatives as a complex socio-psychological phenomenon characterized by specificity and determined by sociocultural, demographic, and personal factors [22]. Other researchers examined characteristic features of the emotional and personal development of adolescents who represent the so-called “forest Nenets” from the taiga area of the Krasnoyarsk Krai [23,24]. A syncretic character, unity, consistency of oneself and the world, and significant structural coherence and uniformity of the world view were pointed out in the structure of self-consciousness of the indigenous representatives of Kamchatka [25].

We should note the limited coverage of the ethnic groups in research dedicated to self-identity development. In particular, there are no studies on personal identity among indigenous adolescents from the Arctic territories of European Russia. We emphasize that the development of an individual as a representative of his/her ethnic group is directly related to the degree of harmonization between sociocultural aspects of the ethnic groups and the multinational environment. The Nenets are a special ethnic community of indigenous peoples in the Arkhangelsk region in the Far North. The Nenets make up less than 0.6% of the total population of the Arkhangelsk region. The majority of the population in the Arkhangelsk region is of Russian nationality (95%). The year 1929 saw the formation of the Nenets Autonomous District, the first national district in the Far North of Russia. Currently, the Nenets Autonomous District is a subject of the Russian Federation within the Arkhangelsk region. It is located in the north of European Russia, mostly beyond the Arctic Circle. The Nenets Autonomous District is home to both indigenous peoples of the Far North of Russia and ethnic Russians. Currently, part of the Nenets people are nomads who practice reindeer herding, and another part (representatives of Russian nationality) live in Naryan-Mar and other urban settlements of the Nenets Autonomous District and the Arkhangelsk region.

This territory is a multi-ethnic socio-cultural environment where representatives of different nationalities live and develop their identity. Thus, it is of particular interest to us to examine the characteristic features of self-identity development among Nenets adolescents.

Having analyzed the above-mentioned approaches, we developed the following hypotheses:

1. There are differences in the components of self-identity between Nenets and Russian adolescents.
2. During adolescence, the self-identity of Nenets adolescents is not static, and the intensity of self-identity components keeps changing.
3. There are differences in self-identity development between Nenets boys and girls.

When testing these hypotheses in this empirical study, we found that there are differences in self-identity components between Nenets and Russian adolescents; during adolescence, the intensity of self-identity components among Nenets adolescents keeps changing, and there are differences in self-identity development between Nenets boys and girls.

## 2. Materials and Methods

### 2.1. Participants

To examine the dynamics of self-identity components among Nenets boys and girls during adolescence and to identify characteristic features of self-identity among Nenets adolescents as compared to the Russian ones, we undertook an empirical study consisting of two stages. During the first stage (from March to May 2018), a group of researchers took a field trip to the city of Naryan-Mar and to the village Krasnoye of the Nenets Autonomous District (Arkhangelsk Region, Russian Federation) to collect empirical data. This is where Nenets adolescents are educated. A specific feature of their education is boarding schools where children who live with their parents in the tundra as nomads are taken by helicopter for the school year (from the beginning of September to the end of May). During this period, the adolescents are separated from their parents and live on the school campus. During the summer holidays, schoolchildren return to their families. Thus, adolescents spend one part of their life in tundra, herding reindeer, living as chums in the traditional environment, and another part in a boarding school in a modern city. It is under these peculiar conditions that Nenets children and adolescents develop their self-identity. For this stage, we selected 41 respondents aged 12–13, of whom 19 were boys and 22 were girls. We also selected 58 respondents—35 boys and 23 girls aged 14–15. All the adolescents were Nenets.

At the second stage of the empirical study (from May to June 2018), we selected adolescents of Russian nationality from the Arkhangelsk region of the Russian Federation. There were 59 respondents at the age of 12–13. Thirty-one of them were boys and 28 were girls. There also were 62 respondents at the age of 14–15. Thirty-two of them were boys and 30 were girls.

The study was initiated by a group of researchers from the Northern (Arctic) Federal University that was named after M.V. Lomonosov. The findings presented in the article are part of a large-scale research aimed at examining the identity of children of different nationalities living in the subarctic territories of the Russian Federation.

### 2.2. Procedure 

At the scoping stage, we prepared a set of documents including information on the relevance of the study, description of the study’s aims, objectives, and stages, as well as characteristics of research methods. This set of documents was submitted to the Ministry of Education and Science of the Arkhangelsk region. As a result, we received authorization to conduct the study.

A number of secondary schools in the Arkhangelsk region were chosen to provide a study base. We met the principals of each participating secondary school and explained the aims, objectives, and the procedure of the study to them. The parents and legal representatives of the students gave us written consent for schoolchildren’s participation in the study. The survey was carried out in groups of 10–12 people each. Before the survey, the boys and girls indicated their age and nationality in writing. The researcher gave the instructions to the students and they filled out the answer sheets. When respondents had questions, the researcher approached them and provided all the necessary explanations individually.

### 2.3. Methods

The survey used a technique developed on the basis of M. Kuhn and T. McPartland’s Twenty Statements Test (TST). The technique is a non-standardized self-description with the free-response answers. It provides a direct measure of an individual’s self-concept. During the survey, the respondents were asked to give twenty different answers to the question “Who am I?” within 12 min. The answers were written spontaneously in the order in which they came to their mind. The analysis took into account the number of characteristics corresponding to each of the self-identity components: gender, social, ethnic, family, professional, individual, physical, interests, and undifferentiated identities. The “gender identity” component included gender (boy, girl, young man, future man, etc.). The “social identity” component included social roles and statuses (schoolchild, citizen, female student, etc.). The “ethnic identity” component included ethnicity (Nenets, Russian, etc.). The “family identity” component included references to kinship and family ties (son, sister, grandson, niece, etc.). The “professional identity” component included professional perspectives and intentions associated with future occupation (auto mechanic, future doctor, programmer, etc.). The “individual identity” component included an indication of one’s own personal qualities and peculiarities of one’s character (kind, bold, insecure, aggressive, etc.). The “physical identity” component included physical characteristics and appearance (tall, beautiful, strong, long-haired, stout, etc.). The “interests identity” component included one’s interests, hobbies, and leisure activities (I like to go for a walk, I love cats, I am a gamer, I play football, etc.). The “undifferentiated identity” component included animated and feature films characters, inanimate objects, and abstract images (Spider-Man, darkness, princess, wolf, etc.). When processing the results of the survey, for each respondent, we counted the frequency of occurrence for characteristics of each self-identity component. Then, in each group of respondents, we calculated average indices in order to identify the intensity degree of one or another self-identity component.

### 2.4. Statistical Analysis

The data were processed using SPSS Statistics 22. To test our hypotheses, the Student’s t-test was applied for each independent sample. With the help of this method, we found significant differences in the intensity degree of self-identity components among Nenets and Russian adolescents. We also found differences in the intensity degree of self-identity components among Nenets adolescents of 12–13 years old as compared to Nenets adolescents of 14–15 years old. In addition, we found differences in the intensity degree of ethnic identity among male and female Nenets adolescents of 12–15 years old.

## 3. Results

Our first hypothesis was that there are differences between self-identity components among Nenets and Russian adolescents. Table 1 shows the intensity degree of self-identity components among Nenets and Russian adolescents.

(1) Statistical analysis showed that such self-identity components as “ethnic identity” (p < 0.001), “professional identity” (p < 0.001), and “family identity” (p < 0.01) are more pronounced among the 12–13-year-old Nenets boys as compared to the Russian ones. At the same time, such components as “individual identity” (p < 0.001) and “undifferentiated identity” (p < 0.01) are less pronounced among 12–13-year-old Nenets boys as compared to the Russian ones. Such self-identity components as “professional identity” (p < 0.001) and “family identity” (p < 0.01) are more prominent among 14–15-year-old Nenets boys as compared to their Russian counterparts. In addition, statistical analysis revealed a tendency for a greater intensity degree of the “ethnic identity” component (p < 0.05) among 14–15-year-old Nenets boys as compared to the Russian ones. The “individual identity” component (p < 0.01) is less pronounced among 14–15-year-old Nenets boys as compared to Russian adolescents. The “undifferentiated identity” component (p < 0.01) is less pronounced among 12–13-year-old Nenets girls as compared to the Russian ones. Statistical analysis also revealed a tendency to a greater intensity degree of the “physical identity” component (p < 0.05) among 12–13-year-old Nenets girls as compared to Russian ones. There were no statistically significant differences in the intensity degree of self-identity components among 14–15-year-old Nenets girls as compared to their Russian counterparts.
(2) Our second hypothesis was that during adolescence. the intensity degree of self-identity components among Nenets adolescents keeps changing. Table 2 shows the dynamics in self-identity components among Nenets girls and boys when they transition from the age of 12–13 to that of 14–15.
  Statistical analysis shows that the intensity degree of the “ethnic identity” component (p < 0.05) has a tendency to decline and the intensity degree of the “individual identity” component (p < 0.05) has a tendency to increase among Nenets boys when they transition from the age of 12–13 to 14–15. When it comes to Nenets girls, the intensity degree of “physical identity” component (p < 0.05) has a tendency to decline while the intensity degree of the “undifferentiated identity” component (p < 0.05) has a tendency to increase when the girls transition from the age of 12–13 to 14–15.
(3) Our third hypothesis was that there are differences in self-identity development among Nenets boys and girls. Table 3 shows differences in self-identity development among 12–15-year-old Nenets boys and girls.

Statistical analysis showed that the “professional identity” component (p < 0.001) and “ethnic identity” component (p < 0.01) are more pronounced among 12–15-year-old Nenets boys as compared to Nenets girls of the same age. The self-identity component of “individual identity” (p < 0.001) is pronounced among 12–15-year-old Nenets boys as compared to Nenets girls of the same age. In addition, 12–15-year-old Nenets boys show a tendency to have a more pronounced “social identity” component (p < 0.05) than Nenets girls of the same age.

## 4. Discussion

The analysis of the results allowed us to answer the questions formulated in our hypotheses. We assumed that there are certain differences between self-identity components among Nenets adolescents and the Russian ones.

The results of self-identity study show that differences in self-identity components among Nenets and Russian adolescents do exist. It should be noted, that these differences are expressed differently among Nenets male and female adolescents. So, when comparing Nenets boys and Russian boys, we see differences in ethnic identity, family identity, professional identity, and individual identity. It is noteworthy that this situation is true for both 12–13-year-old boys and 14–15-year-old ones. Nenets boys, unlike their Russian counterparts, have more pronounced components of ethnic, professional, and family identities, and a less pronounced component of individual identity. Such results show that it is more important for Nenets boys to perceive themselves as members of their ethnic group than it is for their Russian counterparts. They also attach greater importance to their own family role and kinship. Professional perspective and intentions related to their future occupation are more significant for Nenets boys than for the Russian ones. At the same time, when describing themselves, Nenets boys find their own personal qualities and peculiarities of their character less important than their Russian counterparts do. We suppose that such differences have to do with Nenets’ persisting traditional nomadic way of life and life in communities. This leads to an earlier and stable choice of occupation, makes ethnic and family ties more significant while individuality becomes less important. It is noteworthy that 12–13-year-old Nenets boys have a less pronounced undifferentiated component of their self-identity than the Russian ones, that is to say they are much less likely to associate themselves with the characters of animated and feature films, inanimate objects, and abstract images. We assume that this has to do with the nomadic way of life of the Nenets, who spend most of their time in tundra, engaged in such traditional activities as reindeer breeding and hunting.

The intensity degree of self-identity components among Nenets girls differs much less from those of their Russian counterparts. The undifferentiated component of one’s self-identity, for instance, is less prominent among 12–13-year-old Nenets girls as well as among Nenets boys of the same age than among the Russian adolescents. Nenets girls are much less likely to associate themselves with characters from animated and feature films, inanimate objects, and abstract images. It should be noted that 12–13-year-old Nenets girls tend to have a more pronounced “physical identity” component than the Russian ones. That is, Nenets girls attach somewhat greater importance to their physique and appearance. It is noteworthy that the intensity degree of all the self-identity components among 14–15-year-old Nenets girls is similar to that of the Russian girls of the same age. We suppose that such results have to do with the fact that girls are more influenced by social and educational environment in which they live during the school time.

Thus, the differences between self-identity components among Nenets and Russian adolescents turned out to be pronounced among male adolescents. When it comes to female Nenets adolescents, the intensity degree of all of the self-identity components is similar to that of their Russian counterparts. That is, our first hypothesis was only confirmed to a certain extent. Self-identity specifics of different indigenous peoples of Russia as compared to characteristics of self-identity with people of Russian nationality are emphasized in a number of sources [21,22,23,24].

Our next hypothesis was the assumption that during adolescence, the intensity degree of self-identity components keeps changing. Indeed, we see the dynamics in the intensity degree of self-identity components when Nenets adolescents’ transition from the age of 12–13 to that of 14–15. When it comes to Nenets boys, for example, we find changes in such self-identity components as ethnic and individual identities and when it comes to Nenets girls, we see changes in the physical, undifferentiated, and interests identity components. When transitioning from the age of 12-13 to that of 14–15, Nenets boys find it less significant to perceive themselves as members of their ethnic group, while their personal qualities and character peculiarities become more important to them. It should be noted that the intensity degree of these components among both 12–13-year-old Nenets boys and 14–15 years old ones differs greatly from that of their Russian counterparts, but when it comes to the dynamics, these differences gradually becomes smaller. When transitioning from the age of 12–13 to that of 14–15, Nenets girls find their physique and appearance are less important while their interests, hobbies, and leisure activities becomes more significant to them. At the same time, Nenets girls become a little more likely to associate themselves with characters of animated and feature films, inanimate objects, and abstract images. As mentioned above, in terms of the intensity degree of self-identity components, Nenets girls differ very little from the Russian ones. It should be noted that even existing differences in the dynamics have a tendency to gradually decline. Thus, the hypothesis that during adolescence the intensity degree of self-identity components among Nenets keeps changing on the whole was confirmed. The observed changes are a tendency. The dynamics differences in the intensity degree of self-identity components among Nenets boys and girls as compared to the Russian ones tend to gradually decrease. In our opinion, such dynamics in the intensity degree of self-identity components can be explained by the fact that Nenets and Russian adolescents stay a long time in a common socio-educational environment.

Our third hypothesis was that there are differences in self-identity development among Nenets boys and girls. The results of the study show that there are differences in the intensity degree of self-identity components among male and female Nenets adolescents. Nenets boys and girls aged 12–15 years have differences when it comes to the social, ethnic, professional, and individual identity components. Nenets boys have more pronounced professional, ethnic, and social components of self-identity as compared to Nenets girls, while Nenets girls have a more pronounced individual identity component as compared to Nenets boys. That is, Nenets boys find their social roles, professional perspectives, and belonging to their ethnic group more significant, while Nenets girls, on the other hand, attach greater importance to their personal qualities and peculiarities of their character. We suppose that such differences have to do with the fact that the traditional way of life of Nenets peoples is of greater significance for Nenets boys, while Nenets girls are more focused on the conditions and values of the socio-educational environment they live in during the school time. This tendency of indigenous peoples to emphasize their ethnic uniqueness, on the one hand, and adapt to the social environment of the national majority, on the other hand, is confirmed by other researchers [20].

## 5. Conclusions

The results of our study have theoretical and practical value. We proved that there are differences in self-identity components among Nenets and Russian adolescents. During adolescence, the intensity degree of self-identity components among Nenets adolescents keeps changing. There are differences in self-identity development among Nenets boys and girls.

The results of the study contribute to the scientific knowledge about self-identity as a psychological phenomenon. The identified characteristic features of Nenets adolescents will make it possible to design a socio-educational environment for a multi-ethnic space in schools located in the areas inhabited by representatives of different ethnic groups. The knowledge of characteristic aspects of self-identity components development among indigenous adolescents from the Far North will improve the system of psychological and pedagogical support for adolescents.

## Figures and Tables

**Table 1:** Self-identity among Nenets and Russian adolescents.

	Self-Identity Components	Male Sex		Female Sex	
		12–13 years	14–15 years	12–13 years	14–15 years
Nenets adolescents	Gender	0.74 ± 0.15	0.48 ± 0.11	0.68 ± 0.16	0.48 ± 0.13
	Social	4.53 ± 0.44	4.36 ± 0.37	3.05 ± 0.36	4.00 ± 0.51
	Ethnic	0.68 ± 0.16 ***	0.33 ± 0.08 *	0.18 ± 0.09	0.17 ± 0.08
	Family	2.32 ± 0.48 **	2.03 ± 0.32 **	2.05 ± 0.42	2.04 ± 0.35
	Professional	1.84 ± 0.63 ***	1.33 ± 0.21 ***	0.41 ± 0.25	0.30 ± 0.12
	Individual	3.95 ± 0.76 ***	5.79 ± 0.82 **	9.27 ± 0.79	7.83 ± 0.74
	Physical	0.89 ± 0.35	0.52 ± 0.16	1.32 ± 0.31 *	0.57 ± 0.16
	Interests	2.37 ± 0.53	2.18 ± 0.36	1.41 ± 0.36	2.61 ± 0.52
	Undifferentiated	0.11 ± 0.07 **	0.39 ± 0.17	0.05 ± 0.05 **	1.17 ± 0.47
Russian adolescents	Gender	0.49 ± 0.07	0.63 ± 0.09	0.71 ± 0.09	0.67 ± 0.08
	Social	3.48 ± 0.27	4.53 ± 0.32	3.73 ± 0.32	4.35 ± 0.34
	Ethnic	0.21 ± 0.06 ***	0.16 ± 0.06 *	0.09 ± 0.04	0.16 ± 0.05
	Family	1.07 ± 0.19 **	1.09 ± 0.20 **	1.38 ± 0.23	1.59 ± 0.19
	Professional	0.42 ± 0.13 ***	0.47 ± 0.16 ***	0.26 ± 0.13	0.38 ± 0.12
	Individual	7.91 ± 0.57 ***	8.23 ± 0.55 **	8.35 ± 0.53	9.22 ± 0.58
	Physical	0.78 ± 0.12	0.74 ± 0.13	0.79 ± 0.13 **	0.46 ± 0.10
	Interests	2.57 ± 0.26	2.60 ± 0.32	2.05 ± 0.23	2.41 ± 0.28
	Undifferentiated	2.12 ± 0.38 **	0.74 ± 0.25	2.33 ± 0.47 *	0.59 ± 0.24

Note: * *р* ≤ 0.05; ** *р* ≤ 0.01; *** *р* ≤ 0.001.

**Table 2:** Self-identity dynamics among Nenets adolescents when they transition from the age of 12–13 to that of 14–15.

	Self-Identity Components	Male Sex	Female Sex
12–13-year-old Nenets adolescents	Gender	0.74 ± 0.15	0.68 ± 0.16
	Social	4.53 ± 0.44	3.05 ± 0.36
	Ethnic	0.68 ± 0.16 *	0.18 ± 0.09
	Family	2.32 ± 0.48	2.05 ± 0.42
	Professional	1.84 ± 0.63	0.41 ± 0.25
	Individual	3.95 ± 0.76 *	9.27 ± 0.79
	Physical	0.89 ± 0.35	1.32 ± 0.31 *
	Interests	2.37 ± 0.53	1.41 ± 0.36 *
	Undifferentiated	0.11 ± 0.07	0.05 ± 0.05 *
14–15-year-old Nenets adolescents	Gender	0.48 ± 0.11	0.48 ± 0.13
	Social	4.36 ± 0.37	4.00 ± 0.51
	Ethnic	0.33 ± 0.08 *	0.17 ± 0.08
	Family	2.03 ± 0.32	2.04 ± 0.35
	Professional	1.33 ± 0.21	0.30 ± 0.12
	Individual	5.79 ± 0.82 *	7.83 ± 0.74
	Physical	0.52 ± 0.16	0.57 ± 0.16 *
	Interests	2.18 ± 0.36	2.61 ± 0.52 *
	Undifferentiated	0.39 ± 0.17	1.17 ± 0.47 *

Note: * *р* ≤ 0.05; ** *р* ≤ 0.01; *** *р* ≤ 0.001.

**Table 3:** Comparison of self-identity components among 12–15-year-old Nenets boys and girls.

Self-Identity Components	Male Sex	Female Sex
Gender	0.58 ± 0.09	0.58 ± 0.10
Social	4.42 ± 0.28 *	3.53 ± 0.31 *
Ethnic	0.46 ± 0.08 **	0.18 ± 0.06 **
Family	2.13 ± 0.26	2.04 ± 0.27
Professional	1.52 ± 0.26 ***	0.36 ± 0.13 ***
Individual	5.12 ± 0.59 ***	8.53 ± 0.54 ***
Physical	0.65 ± 0.16	0.93 ± 0.18
Interests	2.25 ± 0.29	2.02 ± 0.33
Undifferentiated	0.29 ± 0.11	0.62 ± 0.25

Note: * *p* ≤ 0.05; ** *p* ≤ 0.01; *** *p* ≤ 0.001.
